# Intestinal Parasitic Infections among Pregnant Women in Venezuela

**DOI:** 10.1155/IDOG/2006/23125

**Published:** 2006

**Authors:** Alfonso J. Rodríguez-Morales, Rosa A. Barbella, Cynthia Case, Melissa Arria, Marisela Ravelo, Henry Perez, Oscar Urdaneta, Gloria Gervasio, Nestor Rubio, Andrea Maldonado, Ymora Aguilera, Anna Viloria, Juan J. Blanco, Magdary Colina, Elizabeth Hernández, Elianet Araujo, Gilberto Cabaniel, Jesús Benitez, Pedro Rifakis

**Affiliations:** ^1^Environmental Health, Ministry of Health, Carupano, Sucre 6150, Venezuela; ^2^Center for Parasitological Research JWT, University of Los Andes, Trujillo 3102, Venezuela; ^3^Salud-Miranda, Miranda 1201, Venezuela; ^4^Institute for Pathology, Caracas 1040, Venezuela; ^5^Faculty of Health Sciences, University of Carabobo, Valencia 2001, Venezuela; ^6^Military Hospital, Caracas 1020, Venezuela; ^7^Environmental Health, Ministry of Health, Táchira 5032, Venezuela; ^8^Sanitary District Cagijal, Yaguaraparo, Sucre 6150, Venezuela; ^9^Perez de Leon Hospital, Caracas 1073, Venezuela; ^10^Obstetrics & Gynecology Service, Maracaibo University Hospital, Maracaibo, Zulia 4004, Venezuela; ^11^Chubasquen Hospital, Chubasquen, Portuguesa 3357, Venezuela; ^12^Environmental Health, Ministry of Health, Maracay, Aragua 2102, Venezuela

## Abstract

*Introduction.* Intestinal parasitic infections, especially due to helminths, increase anemia in pregnant women. The results of this are low pregnancy weight gain and IUGR, followed by LBW, with its associated greater risks of infection and higher perinatal mortality rates. For these reasons, in the setting of no large previous studies in Venezuela about this problem, a national multicentric study was conducted. *Methods.* Pregnant women from nine states were studied, a prenatal evaluation with a coproparasitological study. Univariated and multivariated analyses were made to determine risk factors for intestinal parasitosis and related anemia. *Results.* During 19 months, 1038 pregnant women were included and evaluated. Intestinal parasitosis was evidenced in 73.9%: *A lumbricoides *57.0%, *T trichiura *36.0%, *G lamblia *14.1%, *E hystolitica *12.0%, *N americanus *8.1%, *E vermicularis *6.3%, *S stercoralis *3.3%. Relative risk for anemia in those women with intestinal parasitosis was 2.56 (*P* < .01). *Discussion.* Intestinal parasitoses could be associated with conditions for development of anemia at pregnancy. These features reflect the need of routine coproparasitological study among pregnant women in rural and endemic zones for intestinal parasites. Further therapeutic and prophylactic protocols are needed. Additional research on pregnant intestinal parasitic infection impact on newborn health is also considered.

## INTRODUCTION

The soil-transmitted helminthiases are ancient diseases that continue to cause misery and disability in poor populations. The numbers affected are staggering. About 2 billion harbor these infections worldwide, of whom 300 million suffer associated severe morbidity. Of the total number infected, an estimated 400 millions are school-age children. In 1999, World Health Organization (WHO) estimated that schistosomiasis and soil-transmitted helminthiasis represented more than 40% of the disease burden due to all tropical diseases, excluding malaria [1].

Tropical diseases such as malaria, schistosomiasis, intestinal helminths, and filariasis have a dramatic impact on reproductive health. Many cases of unexplained pregnancy loss are due to undiagnosed tropical diseases. Malnutrition or anemia caused by intestinal worms may be worsened by pregnancy and make the pregnancy difficult [2].

In the developing world, young women, pregnant women, and their infants and children frequently experience a cycle, where undernutrition (macronutrient and micronutrient) and repeated infection, including parasitic infections, lead to adverse consequences that can continue from one generation to the next. Among parasitic infections, malaria and intestinal helminths coexist widely with micronutrient deficiencies and contribute importantly to anemia and this cycle of retarded growth and development. In somewhat more limited or focal geographic settings, other parasitic diseases (eg, schistosomiasis, filariasis) contribute similarly to this cycle. It is undoubtedly much better to enter a pregnancy free of infection and nutritionally replete than the various alternatives [3].

Intestinal parasitic infections, especially due to the helminths, increase anemia in pregnant women [2–6]. Additionally, hookworm infections induce deficiencies of iron, total energy, protein, and possible folate and zinc [4, 7]. The results of this are low pregnancy weight gain and intrauterine growth retardation (IUGR), followed by low birth weight (LBW), with its associated greater risks of infection and higher perinatal mortality rates; this has been reported in the majority of studies, although a causal link remains to be completely proven [7–9].

For these reasons, in the setting of no large previous studies in Venezuela about this problem, a national multicentric study was conducted with the objectives to describe preliminarily the epidemiological importance of intestinal parasitosis in pregnant women and its possible impacts.

## METHODS

The study was a transversal analysis of pregnant women attending to prenatal control outpatient health care centers in Venezuela. Pregnant women from fifteen centers located in semi-urban and rural areas of nine states in the country were studied during the period January 2003–July 2004. All women accepted to be studied and included in this study. Women with previous diagnosis of infectious diseases as HIV/AIDS, HBV infection, syphilis, or toxoplasmosis were not enrolled.

Evaluation of those women included, as a part of their routine prenatal control, an initial interrogation, physical examination, and laboratory studies: count of blood cells (CBC) (including thick and thin films, stained with Giemsa), serological screening studies for HIV-1 and -2 (ELISA), HBV (HbsAg and IgM anti-HBc), VDRL, and FTA-ABS, and toxoplasmosis (antibody titers by DAT). For this study, we considered as normal levels of Hb in women those between 12–16 g/dL, and between 37–48% for the hematocrit. An eosinophils proportion up to 4% was considered normal.

All women were asked for a fresh stool sample each for coproparasitological study. The stool samples were masked, coded, and processed for parasitological examination. All stool samples were processed within 2 hours of collection. Isolation of enteric bacterial and viral pathogens was not studied in these samples. Different stool examinations were used for efficacy in detecting parasites. These were direct wet-mount, formaldehyde-ether sedimentation method and modified acid-fast staining techniques [10, 11]. The WHO Guide for Diagnosis of Intestinal Parasitosis (1994) was used as an identification reference [12]. All data were collected with a unique structured questionnaire, and then this information was integrated after being sent by electronic mail to a central database.

Univariated and multivariated analyses were made to determinate risk factors for intestinal parasitosis and related anemia. Differences concerning paraclinical parameters were evaluated using the Fisher exact test and the chi-squared test. Data were analyzed by SPSS for Windows 10.0 and Epi Info v.6.0. All tests were two-sided with differences considered significant at *P* < .05.

## RESULTS

One thousand thirty eight pregnant women were enrolled in this study. The mean age of this population was 25.5 ± 6.5 years old. The mean gestational age at enrollment moment was 28.5 ± 4.0 weeks (60% was on the 3rd trimester).

At clinical evaluations, no apparent significant obstetrical alterations were observed. All women were asymptomatic. All serological studies were negative in all women (HIV, HBV, VDRL, Toxoplasmosis).

Hematological evaluation showed that 65.1% of women presented anemia. The mean hemoglobin levels were 10.3 ± 4.0 g/dL, mean hematocrit was 30.6 ± 1.8%. Eosinophils relative mean proportion was 5.1 ± 4.2%. Eosinophilia was seen in 22.3% women. No other alterations were seen in these women.

Intestinal parasitosis was seen in 767 women (73.9%). From this total, 360 (46.9 %) presented infections due to two simultaneous intestinal parasite species, 84 (10.9 %) with three intestinal parasite species, and only 2 women presented infections due to more than three intestinal parasite species (0.3 %).

In this studied group of women, ten different species of intestinal parasites were found, 2 nonpathogenic protozoans, 3 pathogenic protozoans, and 5 helminths species (Table 1). *Ascaris lumbricoides* was the most common parasite and helminth identified in this study (57.0 %) followed by *Trichuris trichiura* (36.0 %). From those women with dual infection (360), 104 (28.9 %) corresponded to *A lumbricoides* with *Trichuris*.

Univariated and multivariated analyses made to assess risk factors for intestinal parasitosis and related anemia only found significance for the presence of intestinal parasitosis as a risk to have anemia during pregnancy, relative risk (RR) was 2.56 (95%CI 2.13–3.08) (Table 2). When this was adjusted for helminth infection, RR = 1.56 (95%CI 1.43–1.69), and for protozoan infection, RR = 1.49 (95%CI 1.38–1.61). Additionally relative risk was adjusted for helminth species; for *A. lumbricoides*, RR = 2.01 (95%CI 1.83–2.20); *trichiura*, RR = 1.18 (95%CI 1.08–1.30); *Necator americanus*, RR = 1.23 (95%CI 1.07–1.41). Finally, relative risk was also adjusted for dual helminth infections and those due to *A lumbricoides* and *T trichiura*, RR = 1.99 (95%CI 1.83–2.15) and RR = 1.60 (95%CI 1.51–1.70), respectively (Table 2). No association was made with any other variable evaluated.

Finally when compared, levels of hemoglobin, hematocrit, and eosinophils count were significantly different between those women with intestinal parasitic infections (Table 3). With the eosinophilia, we found significance for its presence as marker to be diagnosed as to have intestinal parasitosis during pregnancy (27.6% in infected women, 7% in noninfected women), RR was 1.33 (95%CI 1.26–1.42; χ^2^_Yates _ = 48.07, *P* < .0001).

## DISCUSSION

The burden of disease imposed on helminth-infected girls and women in childbearing age, especially when pregnant, may very well define the single most important contribution of intestinal parasitic infections to the calculation of their global disease burden. Pregnancy requires extra nutrients, especially iron, and produces a “physiological anemia” due to hemodilution [13, 14]. The anemia results in both decreased appetite and lowered aerobic and physical work capacity, even without the added weight gain to transport—and this is in girls and women who, in the tropics, must often carry their youngest child and the household water supply long distances and also manage to till their fields daily without mechanized equipment. The total amount of work a woman can do in a day definitely decreases when she is anemic, whatever the cause is, and pregnancy plus helminth infections produce a double burden for women in some rural farming communities. [7] Women may even acquire helminth infections in the process of growing the family's food and thus increase their degree of anemia in pregnancy, as for example, in Vietnam, where insufficiently composted human feces may be used as fertilizer on vegetable crops [15].

In this study, where more than a thousand young asymptomatic pregnant women were evaluated, we observed a high prevalence of intestinal parasitosis (more than 70%), higher than those previously reported for pregnant women in Congo (9%) [6], Nigeria (12.5%) [5], Mexico (38.2%) [16], Brazil (Sao Paulo State, 45.1%; Rio de Janeiro State, 69.2%) [17, 18], and Indonesia (69.7%) [4]. Although current evaluated women were transversally analyzed and more than 65% had anemia, those with anemia corresponded mainly to infected women (more than 80% of those with anemia). Almost the half of infected women presented mixed infections, due to two different parasite species which represented a significant risk to have anemia, almost twice than those women who did not. But the most important risk factor to be found with anemia in pregnancy at this series was to be simply presented an intestinal parasitic infection at pregnancy (RR = 2.56), independently if this was due helminths or protozoans. Species adjusted analysis showed that for this study the most important parasite representing a risk to be found with anemia at pregnancy was *A lumbricoides *(RR = 2.01); the risk of anemia has been frequently reported for *N americanus*, *Ancylostoma duodenale, T trichiura*, *Strongyloides stercoralis *and *Enterobius vermicularis* [7, 19, 20], but rarely for *A lumbricoides* [21], which may cause intestinal obstruction, liver abscess, local irritation, and damage with malabsorption as main cellular related events associated with the infection [21, 22]; *A lumbricoides* plays an important role in precipitating protein-energy malnutrition in undernourished children [23], this infectious disease is a form of malnutrition [21]. Those women infected presented not just a higher frequency of anemia but also significant lower levels of hemoglobin and hematocrit and, obviously, higher levels of eosinophilia. This last, at least in this series, evidenced to be a diagnostic marker for intestinal parasitosis, and with it, this type of infection in pregnant women at endemic zones could be suspected, even if a stool screening is negative (which should be repeated at least 3 consecutive times). Intestinal parasites (especially helminths) can be tissue dwelling or intestinal but all induce a dramatic expansion of the Th2 lymphocyte subset [24, 25]. It remains unclear whether these Th2-derived responses, including IgE, eosinophilia, and mastocytosis are important in the protective immune response to the parasite, or are responsible for immune-mediated pathology, or both [24], but at least is a paraclinical marker of infection.

 Given these results, the importance and potential impacts of intestinal parasitosis at pregnancy, such as the anemia, are quietly obvious. Independently to etiology, parasitoses are associated with conditions for development of anemia at pregnancy [7]. This indicates the need for periodical stool examinations during pregnancy as part of routine laboratory test in the prenatal control of women. Considering this, systematic screening and treatment of anemia and associated factors such as intestinal helminthiasis and protozoasis is needed for the pregnant women population, as has been established in other countries [6], to significantly improve the health of mothers and children, because it is undoubtedly much better to enter a pregnancy free of infection and nutritionally replete than the various alternatives. Existing intervention strategies for micronutrient support and for the control of common parasitic infections before or during pregnancy, particularly intestinal parasitosis, should be followed. However, further research to identify barriers and priority approaches to achieving this goal remain very important in resource-poor settings, where targeted public health efforts are required [3].

As has been stated in other studies, it is necessary to modify some preventive measures of information and education and to give specific treatment before the pregnancy in order to increase some of the pregnant women's health indicators. The newborn of mothers with intestinal parasitosis have a greater probability of being born with less weight than what is expected [16], although we did not evaluate this issue on the current study.

These results enhanced the need for wide implementation of intermittent iron and folic acid supplementation as a valid strategy when used as a preventive intervention in prenatal care settings [14]. But that antihelminthic therapy could be given to infected women before conception as public health strategy to improve iron status may be also considered. Additionally once diagnosed, if the woman is on the second or third gestation trimester, it could be treated. In the current study, all women in this condition were treated with mebendazol 500 mg after first trimester, as has been recommend by some authors who consider a possible national strategy for prevention and control is to give a single dose of this drug during the second or third gestation trimesters [26].

The current study reflects the need of routine coproparasitological study among pregnant women in rural and endemic zones for intestinal parasites. Even more, further therapeutic and prophylactic protocols are needed. Additional research on pregnant intestinal parasitic infection impact on newborn health is on-going.

## Figures and Tables

**Table 1:** Parasite positivity in stool specimens examined from pregnant women studied.

	Number	(%)

Protozoans		
Nonpathogenic		
*Entamoeba coli*	44	5.7
*Endolimax nana*	30	3.9
Pathogenic		
*Giardia lamblia*	108	14.1
*Entamoeba histolytica/dispar*	92	12.0
*Cryptosporidium spp*	2	0.3
Helminths		
*Ascaris lumbricoides*	437	57.0
*Trichuris trichiura*	276	36.0
*Necator americanus*	62	8.1
*Enterobius vermicularis*	48	6.3
*Strongyloides stercoralis*	25	3.3

**Table 2:** Relative risk for anemia at pregnancy according to the presence of intestinal parasitosis.

Variable (risk for anemia)		Normal			
	Anemia	Hb	RR	χ^2^_Yates_	*P*

Intestinal parasitosis at pregnancy					
Present	594	173	2.56	194.24	< .0001
Absent	82	189	—	—	—
Helminth infection at pregnancy					
Present	322	61	1.56	94.63	< .0001
Absent	354	301	—	—	—
Protozoan infection at pregnancy					
Present	179	23	1.49	59.65	< .0001
Absent	497	339	—	—	—
*Ascaris lumbricoides *infection					
Present	401	36	2.01	233.76	< .0001
Absent	275	326	—	—	—
*Trichuris trichiura *infection					
Present	203	73	1.18	11.25	.0008
Absent	473	289	—	—	—
*Necator americanus *infection					
Present	49	13	1.23	4.98	.2565
Absent	627	349	—	—	—
Dual helminth infection					
Present	347	13	1.99	235.08	< .0001
Absent	329	349	—	—	—
*A lumbricoides *+ *T trichiura*					
Present	106	2	1.60	56.27	< .0001
Absent	570	360	—	—	—

Total	676	362	—	—	—

**Table 3:** Comparisons between levels of hematological laboratory variables in those women with or without intestinal parasitosis (mean ± SD).

	(*n* = 767) Infected	(*n* = 271) Noninfected	F	*P*

Hemoglobin (g/dL)	9.6 ± 0.7	11.7 ± 0.4	2185.89	< .0001
Hematocrit (%)	29.9 ± 1.2	34.7 ± 1.8	2416.68	< .0001
Red cells count M (×10^6^/mm^3^)	4.1 ± 0.9	4.2 ± 0.2	3.29	.0701
White cells count (×10^3^/mm^3^)	7.5 ± 2.9	7.8 ± 2.2	2.41	.1209
Neutrophils (%)	52.3 ± 5.8	53.0 ± 5.5	3.00	.0837
Lymphocytes (%)	33.6 ± 8.9	34.2 ± 7.7	0.97	.3239
Eosinophils (%)	8.9 ± 4.9	1.1 ± 3.7	571.43	< .0001
Platelets (×10^3^/mm^3^)	212.8 ± 28.8	216.2 ± 36.2	2.42	.1197
